# The Delayed Risk Stratification System in the Risk of Differentiated Thyroid Cancer Recurrence

**DOI:** 10.1371/journal.pone.0153242

**Published:** 2016-04-14

**Authors:** Aldona Kowalska, Agnieszka Walczyk, Iwona Pałyga, Danuta Gąsior-Perczak, Klaudia Gadawska-Juszczyk, Monika Szymonek, Tomasz Trybek, Katarzyna Lizis-Kolus, Dorota Szyska-Skrobot, Estera Mikina, Stefan Hurej, Janusz Słuszniak, Ryszard Mężyk, Stanisław Góźdź

**Affiliations:** 1 Departments of Endocrinology, Holycross Cancer Centre, Kielce, Poland; 2 Department of Surgical Oncology, Holycross Cancer Centre, Kielce, Poland; 3 Department of Cancer Epidemiology, Holycross Cancer Centre, Kielce, Poland; 4 Department of Clinical Oncology; Holycross Cancer Centre, Kielce, Poland; 5 The Faculty of Health Sciences, Jan Kochanowski University, Kielce, Poland; IPATIMUP/Faculty of Medicine of the University of Porto, PORTUGAL

## Abstract

**Context:**

There has been a marked increase in the detection of differentiated thyroid carcinoma (DTC) over the past few years, which has improved the prognosis. However, it is necessary to adjust treatment and monitoring strategies relative to the risk of an unfavourable disease course.

**Materials and Methods:**

This retrospective study examined data from 916 patients with DTC who received treatment at a single centre between 2000 and 2013. The utility of the American Thyroid Association (ATA) and the European Thyroid Association (ETA) recommended systems for early assessment of the risk of recurrent/persistent disease was compared with that of the recently recommended delayed risk stratification (DRS) system.

**Results:**

The PPV and NPV for the ATA (24.59% and 95.42%, respectively) and ETA (24.28% and 95.68%, respectively) were significantly lower than those for the DRS (56.76% and 98.5%, respectively) (p<0.0001). The proportion of variance for predicting the final outcome was 15.8% for ATA, 16.1% for ETA and 56.7% for the DRS. Recurrent disease was rare (1% of patients), and was nearly always identified in patients at intermediate/high risk according to the initial stratification (9/10 cases).

**Conclusions:**

The DRS showed a better correlation with the risk of persistent disease than the early stratification systems and allows personalisation of follow-up. If clinicians plan to alter the intensity of surveillance, patients at intermediate/high risk according to the early stratification systems should remain within the specialized centers; however, low risk patients can be referred to endocrinologists or other appropriate practitioners for long-term follow-up, as these patients remained at low risk after risk re-stratification.

## Introduction

The incidence of differentiated thyroid carcinoma (DTC) is rapidly increasing. Over the past 30 years, the incidence in the United States has tripled from 4.9 to 14.3 per 100,000 inhabitants [1]. Similar trends have been reported in other countries [2–5]. It is estimated that, by 2019, DTC will be the third most common cancer in women [6]. Papillary thyroid carcinoma (PTC) accounts for almost 90% of new cases, with 39% of primary tumours measuring less than 10 mm in diameter [7,8]. The prognosis for PTC is very good: 99% of patients with stage I–III tumours survive for 10 years after diagnosis. However, there is a risk of recurrence, and distant metastases have been identified 40 years after the initial diagnosis; therefore, lifelong surveillance is recommended [9]. Current adjuvant treatment protocols and remission status monitoring methods place a great burden on patients; this is due to exposure to ionising radiation via therapeutic and diagnostic use of ^131^I, periods of hypothyroidism, exposure to suppressive doses of levothyroxine (LT4) and frequent follow-up appointments [10,11]. The marked increase in the rate of early stage carcinomas over the past few years required a different management strategy [12]. It is necessary to adjust treatment protocols and the intensity of oncologic surveillance relative to the risk of an unfavourable disease course. The Union for International Cancer Control-American Joint Committee on Cancer (UICC-AJCC) staging system for thyroid carcinoma, which is based on histopathology results and the patient’s age, shows a good correlation with the risk of mortality; however, it does not predict the risk of recurrence [13]. The stratification systems recommended by the American Thyroid Association (ATA) and the European Thyroid Association (ETA) show a better correlation with disease course, but are based solely on data obtained shortly after surgery and do not take into account changes during follow-up [10,11]. More recently, the superiority of a new system proposed by Tuttle [14], called the “ongoing risk stratification” system, has become clear. This system takes account of changes in the initial risk level according to data obtained after completion of initial treatment. In 2011, Castagna et al., reviewed the system proposed by Tuttle, and called it the Delayed Risk Stratification System. The new system has also been validated by other authors [15–20].

The aim of the present study was to compare the utility of the delayed risk stratification (DRS) system for predicting the clinical course and for planning patient monitoring strategy with that of the systems currently recommended by the ATA and ETA.

## Materials and Methods

### Patients and study design

A total of 916 consecutive patients with a histopathologically confirmed diagnosis of DTC and who had completed initial treatment (total thyroidectomy followed by adjuvant ^131^I treatment) at a single centre between 2000 and 2013 were enrolled in the study. Patients with early stage tumours, those who had undergone resection of a single lobe plus the isthmus only, those who had not received ^131^I treatment following total thyroidectomy and those diagnosed with a pT1aN0 DTC after undergoing partial thyroidectomy for other reasons were excluded.

The study plan was accepted by the Bioethics Committee at the Regional Chamber of Physicians without the necessity to obtain the patients’ written informed consents as the data obtained was retrospective data from the patients‘ medical history that was carried out during routine diagnostic procedures while hospitalised. All patients' records/information were anonymized and de-identified prior to analysis.

### Treatment protocol

The treatment protocol involved total thyroidectomy with central compartment lymph node dissection, followed by adjuvant treatment with ^131^I and suppressive doses of LT4. Treatment with ^131^I was preceded by 2 weeks on a low-iodine diet and was performed after endogenous TSH stimulation (TSH >30 mIU/l; without thyroid hormone, starting from the time of thyroidectomy). Neck sonography was performed, and thyroglobulin (Tg) and anti-Tg levels were measured, and a whole body scan (WBS) was undertaken at 5 days post-treatment. The tumours were staged according to the UICC-AJCC TNM staging system (7th edition). N0 status was established according to histopathology or, if histopathological examination of post-operative specimens did not suggest lymph node involvement, N0 status was established by clinical assessment based on sonography and fine-needle aspiration biopsy (FNAC) followed by measurement of Tg in the aspirate. Patients were classified into persistent/recurrent disease risk groups according to the systems recommended by the ATA and ETA [10,11]. The efficacy of the initial treatment was assessed at 9–12 months after treatment with ^131^I. Follow-up assessment of 563 patients was performed after endogenous TSH stimulation, which required interruption of LT4 therapy for 4 weeks. A total of 352 patients underwent stimulation with recombinant human (rh)TSH. The levels of stimulated Tg and anti-Tg were measured in all patients, and neck sonography and WBS were performed. Patients with failed ablation, defined as the presence of focal ^131^I uptake in the neck with thyroid bed uptake of >0.1% (n = 86), received a second dose of ^131^I and were re-evaluated 9–12 months later.

### Assessment of treatment response

Response to treatment was assessed according to the criteria proposed by Momesso et al., (excellent response, biochemical incomplete response, structural incomplete response and indeterminate response) [21]. Patients with an excellent response were classified as low risk (LR) according to the DRS, while the remaining patients were classified as high risk (HR). Disease evolution was monitored during follow-up visits by measuring Tg levels during LT4 treatment (Tg/LT4), by measuring stimulated Tg levels, and by neck sonogram, WBS, and measurement of anti-Tg antibodies. Between 2000 and 2010, Tg production was stimulated by discontinuing LT4 treatment for 4 weeks, and from 2011 onward by administering rhTSH. No evidence of disease (NED) was defined as follows: a normal neck sonogram; lack of ^131^I uptake on WBS; a Tg/T4 ratio <1.0; a Tg/THW ratio <2.0; and, if anti-Tg were present, a decline in levels during follow-up. Biochemically persistent disease was defined as a Tg/T4 ratio ≥ 1.0, a Tg/rhTSH ratio ≥ 1.0, or a Tg/TWD ratio ≥ 2.0, with no evidence of disease (NED) on imaging. Structurally persistent disease was defined as the presence of neoplastic changes on ultrasound or WBS.

Recurrent disease was defined as biochemical or structural evidence of disease after a period of NED.

### Investigations

Tg concentrations were measured using the Immulite 2000 xpi Immunoassay System analyser (Siemens Heatlhcare Diagnostics, United Kingdom). The method has an analytical sensitivity of 0.2 ng/ml and a functional sensitivity of 0.9 ng/ml.

Basal serum samples taken from each patient were screened for the presence of Tg antibodies using the Immulite 2000 xpi Immunoassay System analyser, with an analytical sensitivity of 2.2 IU/ml. Neck ultrasonography was performed using a Siemens Versa pro and a Hitachi EUB-6500 (both featuring a colour Doppler function), with a high frequency linear probe (7.5 MHz). WBS was performed with a Symbia T2 gamma camera (Siemens) using a high energy collimator with a scanning speed of 10 cm/min. Diagnostic WBS was performed 72 hours after the administration of 180 MBq ^131^I (rhTSH) or 80 MBq ^131^I (TWD). Post-therapy WBS was performed on Day 5 after radioactive iodine treatment.

### Statistical analysis

Continuous data are expressed as the mean and standard deviation, or as the median and inter-quartile range. Categorical data were presented as numbers of patients and percentages. Pearson's chi-square test was applied to evaluate significant differences in data frequency.

Diagnostic accuracy, expressed as the positive predictive value (PPV) and negative predictive value (NPV), and 95% confidence intervals (CI) were calculated.

Nagelkerke’s estimation of proportion of variance explained (PVE%) was calculated by logistic regression analysis. All statistical analyses were performed using MedCalc Statistical Software, version 15.6.1 (MedCalc Software bvba, Ostend, Belgium; https://www.medcalc.org; 2015). A p-value <0.05 was considered statistically significant.

## Results

Table 1 shows the clinical characteristics of the patients. The mean age was 51.6±14, and 84.7% were women. PTC was the dominant histology (89.1%), and the tumour was multifocal in 26% of patients. Lymph node involvement at diagnosis was evident in 15.9% of cases. Most patients (62%) had stage I tumours. According to the 2009 ATA classification, the percentage of patients in the LR, intermediate risk (IR) and HR groups was 59.6%, 34.6% and 5.8%, respectively. Distant metastases at diagnosis were evident in 2.8% of patients. According to the 2006 ETA classification, 20.9%, 37.3% and 41.8% of patients were considered very LR, LR or HR, respectively. Patients who had undergone surgery received adjuvant ^131^I treatment (1100–3700 MBq, depending on TNM status). All the patients underwent WBS at 5 days after radionuclide treatment.

**Table 1:** Characteristics of the 916 differentiated thyroid cancer patients.

	Characteristic	Values	Number of patients
**Age (years)**	-Mean ± SD	51.6±14	
	-Median	53	
	-Range	15–89	
**Gender**	-Male	15.3%	140
	-Female	84.7%	776
**Histology**	-Papillary	89.1%	816
	-Follicular	6.7%	61
	-Hürthle cell	1.5%	14
	-Poorly differentiated	2.7%	25
**AJCC stage**^**a**^	-I	62%	568
	-II	8.3%	76
	-III	23.7%	217
	-IV	6.0%	55
**ATA initial risk classification**^**b**^	-LR^c^	59.6%	546
	-IR^c^	34.6%	317
	-HR^c^	5.8%	53
**ETA initial risk classification**^**b**^	-Very LR^c^	20.9%	191
	-LR^c^	37.3%	342
	-HR^c^	41.8%	383
**Lymph node involvement**	-N0	84.1%	770
	-N1	15.9%	146
**Distant metastasis**	-M0	97.2%	890
	-M1	2.8%	26
**Multifocality**	-Yes	26.3%	241
	-No	73.7%	675
**Response to therapy classification**	-Excellent	79.8%	731
	-Indeterminate	10.5%	96
	-Biochemically incomplete	3.3%	30
	-Structurally incomplete	6.4%	59
**Recurrent disease**	-LR^c^	0,2%	1
	-HR^c^	3,73%	9
**Follow-up duration**	-Mean ± SD	7±4	916
	-Median	7	
	-Range	1–13	
**Status of final follow-up**	-No evidence of disease	83.63%	766
	-Persistent disease	12%	110
	-Tumour-related deaths	1.86%	17
	-Tumour-unrelated deaths	2.51%	23

^a^AJCC, American Joint Committee on Cancer

^b^ATA, American Thyroid Association; ETA, European Thyroid Association

^c^LR, low risk; IR, intermediate risk; HR, high risk.

### Response to initial treatment

Treatment efficacy was evaluated in all patients at 9–12 months after ^131^I treatment. An excellent response was identified in 731 patients (79.8%), all of whom were considered disease-free. Biochemical and structural incomplete responses were identified in 30 (3.3%) and 59 patients (6.4%), respectively. The response was undetermined in 96 patients (10.5%).

### Recurrent disease

Patients were followed up for an average of 7 years (range, 1–13 years). Recurrent disease was identified in ten patients (1%) only. Table 2 shows the clinical characteristics of the patients with recurrent disease. Only one (a female) patient with recurrent disease was classified as LR at baseline according to the ATA and ETA, whereas the remaining ten had been classified as IR or HR (despite an excellent response to initial therapy). Recurrence was diagnosed after an average of 7 years. Two patients developed lung metastases (visualised by FDG PET), three developed nodal recurrence in the neck (diagnosed by ultrasound and confirmed by FNAC followed by measurement of Tg in the aspirate) and one developed recurrence in the thyroid bed (diagnosed by ultrasound and confirmed by FNAC and measurement of Tg in the aspirate). ^131^I uptake was identified in the thyroid bed in two patients and in the mediastinum in two patients.

**Table 2:** Clinical characteristics of ten patients with recurrent disease after successful initial therapy.

Patient	Gender^a^	Age	Histology^b^	TNM stage	Time of recurrence (years)	Type of recurrence	Second therapy	Follow-up^c^
TC	F	58	Fol.	T2N1	8	Structural pulmonis	^131^I CHTH	PS
KA	F	60	Pap.	T3N1	4	Structural cervical uptake of ^131^I	^131^I	NED
TD	F	75	Pap.	T1N0	10	Structural mediastinal uptake of ^131^I	^131^I	PS
EG	F	48	Pap.	T3N0	8	Structural cervical uptake of ^131^I	^131^I	NED
MJ	F	41	Ins.	T1bN0	2	Structural recurrence in the thyroid bed	Surgery	NED
JN	F	62	Pap.	T3N0	10	Structural: Lungs	^131^I	PS
AS	F	45	Pap.	T1bN1	2	Structural: Lymph nodes	Surgery	NED
KT	F	58	Pap.	T3mN1	4	Structural: Lymph nodes	Surgery	NED
ZZ	F	52	Pap.	T2N1	13	Structural: Lymph nodes	Surgery	NED
MD	F	68	Pap.	T3N0	7	Biochemical: Tg/rhTSH (6.8 ng/ml)	^131^I	PD

^a^Gender: F, female

^b^Histology: Fol., follicular; Pap.,papillary; Ins, insular

^c^Follow-up: PS, persistent structural disease; NED, no evidence of disease; PD, persistent biochemical disease.

### Evaluation at the end of the follow-up period

At the end of the follow-up period, 766 patients (83.63%) were classified as NED, 100 (12%) had persistent disease, and 40 (4.37%) had died (with 17 [1.86%] of the deaths being tumour-related and the other 23 [2.51%] being tumour-unrelated). All the patients that died from thyroid carcinoma were classified as HR at baseline. Ten of these (56%) had distant metastases at diagnosis. None of the patients entered remission as a result of initial treatment; the disease was considered structurally persistent in 11 patients and biochemically persistent in six.

### Correlation between the recurrent/persistent disease risk stratification systems and the course of disease

The correlation between the ATA, ETA and DRS risk stratification systems and clinical course (NED, persistent disease, recurrent disease or death) is shown in Table 3. For the sake of clarity, patients were classified in two risk groups within each classification system as follows: (1) ATA/LR and HR (IR and HR combined); (2) ETA/LR (very LR and LR combined) and HR; and (3) DRS/LR (excellent response) and HR (indeterminate response, biochemical incomplete response and structural incomplete response combined). According to the ATA and ETA, 95.43% and 95.69%, respectively, of patients in the LR group were classified as NED at the end of follow-up, while the DRS classified 98.6% of patients in the LR group as NED (p<0,001). The ATA and ETA classified 75.6% and 75.98%, respectively, of patients in the HR group as NED at the end of follow-up, whereas the DRS classified only 43.24% in this group as NED (p<0,001). We then examined the utility of the three risk stratification systems for predicting clinical course by calculating the PPV, NPV and PVE% values. The results are shown in Table 4. The PPV for the ATA and ETA systems was very low (24.59% and 24.28%, respectively); however, that for the DRS was much higher (56.76%) (p<0.0001 for ATA and ETA *vs*. DRS). All three systems showed a very high NPV (95.42%, 95.68% and 98.5% for ATA, ETA and DRS, respectively; p>0.05). On the other hand, the PVE% for the ATA and ETA was 15.8% and 16.1%, respectively, and that for the DRS was 56.7% (p<0.0001 for ATA and ETA *vs*. DRS). Taken together, these results show that the DRS system is superior to the ATA and ETA systems.

**Table 3:** Clinical outcome at the end of follow-up according to the ATA, ETA and DRS systems.

Stratification type	NED^a^	Persistent biochemical disease	Persistent structural disease	Recurrent disease	Tumour-related deaths
ATA					
LR: 547 (59.71%)	522 (95.43%)	15 (2.74%)	9 (1.65%)	1 (0.18%)	0
HR: 369(40.29%)^b^	279 (75.61%)	41 (11.11%)	23 (6.23%)	9 (2.44%)	17 (4.61%)
ETA					
LR: 533 (58.19%)	510 (95.69%)	15 (2.81%)	7 (1.31%)	1 (0.19%)	0
HR: 383(41.81%)^b^	291 (75.98%)	43 (11.23%)	23 (6.0%)	9 (2.35%)	17 (4.44%)
DRS					
LR: 731 (79.8%)	721 (98.6%)	0	0	10 (1.2%)	0
HR: 185 (20.2%)^c^	80 (43.24%)	58 (31.35%)	30 (16.22%)	0	17 (9.19%)

^a^NED, no evidence of disease

^b^ATA, American Thyroid Association; ETA, European Thyroid Association

^c^DRS, delayed risk system.

**Table 4:** PPV, NPV and PVE according to the different risk stratification systems.

Stratification type	NPV%^a^ (95% CI)	PPV%^b^ (95 CI)	PVE%^c^
**ATA**	95.42	24.59	15.8
	(93.31–97.02)	(20.29–29.31)	
**ETA**	95.68	24.28	16.1
	(93.60–97.25)	(20.07–28.90)	
**DRS**	98.50	56.76	56.7
	(97.32–99.25)	(49.29–64.01)	

ETA *vs*. ATA, p = 0.9065; ETA *vs*. DRS, p<0.0001; ATA *vs*. DRS, p<0.0001.

^a^PPV, positive predictive value.

^b^NPV, negative predictive value.

^c^PVE, proportion of variance explained.

The risk of recurrence in DTC patients was very low (1%). Recurrent disease developed in patients that were classified as IR/HR at baseline according to the ATA and ETA. These were patients with T3 tumours (infiltration outside the thyroid) or with N1 status, or patients with unfavourable histology (poorly differentiated tumours). Only one patient with recurrent disease was classified as LR at baseline; however, no lymph node involvement was identified upon histopathological examination (Nx), and 10 years later she showed increased Tg levels and mediastinal ^131^I uptake. These findings suggest that the three risk stratification systems are useful for predicting an unfavourable clinical course (persistent or recurrent disease). The DRS identifies patients with persistent disease who require further evaluation and, possibly, additional treatment, while a combination of the three systems assists in deciding the monitoring strategy. Patients showing excellent responses to initial treatment and classified as LR at baseline (491 patients [53.6%]) were practically free of any risk of recurrence (0.2%). A higher risk of recurrence (3.73%; nine cases of recurrent disease among 241 patients classified as HR according to the ATA and who had completed initial treatment in the LR group) was observed among patients who showed an excellent response to initial treatment but were classified as HR; it is these patients that require closer surveillance (241 patients [26.3%]).

## Discussion

The increasing incidence of DTC and the higher percentage of early stage tumours among new cases require changes to both treatment and monitoring. At many cancer centres, patients with DTC remain under lifelong surveillance, despite being classified as NED for many years. Personalisation with respect to the type and frequency of monitoring, depending on the risk of recurrence, is therefore needed. Various recurrence risk assessment systems are being evaluated. The risk stratification systems recommended by the ATA and ETA allow the accurate identification of patients at LR of recurrent and persistent disease. Here, we found that the NPV for both of these systems was similar (95.4% and 95.68%, respectively). Indeed, Castagna et al. [18] obtained similar results (90.6% and 91.3%, respectively). Both systems are, however, characterised by unsatisfactorily low PPV values (24.59% and 24.28%, respectively, in the present study). This is because a large group of patients were classified as IR/HR but remained NED at the end of the follow-up (75.6% and 75.9%, respectively). These results are again consistent with those reported by Castagna et al., who demonstrated that about 60% of patients at IR/HR were NED at the end of follow-up, with PPV values for both systems being 39.2% and 38.4%, respectively [18]. When we compared the utility of the ATA and ETA systems with that of the DRS system, we found that the DRS was superior (PPV for ATA, 15.8%; PPV for ETA, 16.1%; PPV for DRS, 56%; p<0.0001). Again, these values are similar to those reported by Tuttle et al. (values calculated 2 years after the completion of initial treatment) [15] and Castagna et al. (values calculated 8 to 12 months after completion of initial treatment) [18]. A notable finding of the present study was a low rate of recurrence (1%). Nearly all cases of recurrence were observed in patients with T3 tumours, N1 status, or aggressive histology, i.e., patients classified as IR/HR at baseline. This is consistent with a report by Nascimento et al., who showed 1% of recurrence (patients with aggressive histology or T3N1 tumours) [22]. Ito et al. showed a correlation between the rate of recurrence and tumour size: the risk of recurrence of T2 and T3 tumours was four and six times higher, respectively, than that of T1 tumours [23]. Tuttle et al. reported that the risk of recurrence (1.36%) did not differ significantly between LR, IR and HR patients [15]. However, Scheffel et al. [24] reported the risk of recurrence as 2.8%, with biochemical recurrence accounting for as many as 80% of cases, and nodal recurrence accounting for a mere 20%. Recurrent disease was identified in patients who were classified as LR (30%), IR (50%) or HR (20%) at baseline; however, the authors failed to identify any factor(s) that would allow the prediction of recurrent disease. In our study, neither patients with an indeterminate response to initial treatment and whose Tg levels subsequently progressed to values meeting the criteria for biochemically persistent disease, nor patients diagnosed with structural disease, were classified as recurrent disease; rather, they were classified as biochemically or structurally persistent disease. This is in contrast to the approach taken by Castagna et al., who included recurrent disease in the HR group according to the DRS; hence, they reported a higher recurrence rate (1.9%) [18]. An important finding is the importance of neck ultrasound as a method that allows imaging of recurrent foci, both in the thyroid bed and in the cervical lymph nodes. Up to 40% of recurrences in the present study (three nodal recurrences and one recurrence in the thyroid bed) were detected using this method. Han et al. [25] also highlighted the importance of ultrasound for detecting recurrent disease (with 11 out of 13 recurrences being reported as nodal recurrence visible on sonograms). The results reported herein justify a change in the management strategy for patients with DTC. Patients at LR of recurrent/persistent disease according to early stratification and who have shown excellent responses to initial treatment are practically free of any risk of recurrence; these patients may be discharged from the cancer facility. However, patients initially classified as HR, despite excellent responses to initial treatment (e.g., those for whom the risk of recurrence exceeds 3%), and all patients with persistent disease, should undergo continued oncologic surveillance.

## Conclusions

Systems of early and delayed stratification of the risk of recurrent/persistent disease are useful for planning and monitoring treatment and disease course. The DRS showed a better correlation with the risk of persistent disease than the early stratification systems. The risk of recurrence was generally very low (1% in our study) and, in practical terms, was only present in patients classified as having an excellent response to initial treatment but, according to early stratification, were placed in the IR/HR group. When planning changes to the intensity of surveillance, patients classified as IR/HR by early stratification methods should remain in the specialized centers and undergo periodic sonographic and Tg monitoring; however, the care of LR patients that show an excellent response to initial treatment may be referred to endocrinologists or other appropriate practitioners for long-term follow-up.
